# Impaired Autophagy Causes Severe Corneal Neovascularization

**DOI:** 10.3390/cells11233895

**Published:** 2022-12-02

**Authors:** Kun Yi, Yuping Yang, Ye Yuan, Yingqian Xiang, Shanbi Zhou

**Affiliations:** Chongqing Key Laboratory of Ophthalmology and Chongqing Eye Institute, The First Affiliated Hospital of Chongqing Medical University, Chongqing 400016, China

**Keywords:** corneal neovascularization, autophagy, macrophage polarization, alkali burn

## Abstract

Purpose: To investigate the role of macrophage autophagy in the process of corneal neovascularization (CNV). Methods: In vivo, mice CNV was induced by alkali injury and compared with rapamycin-treated alkaline burn mice. Western blot was used to determine the autophagic status of the macrophages. We quantified the levels of macrophage polarization markers (CD86, INOS, CD163, CD206) by RT-qPCR and measured inflammatory factors through ELISA (IL-6 and TNF-α) in the early phase after injury. In vitro, the human umbilical vein endothelial cells (HUVECs) were co-cultured with macrophage-conditioned medium (MCM) induced by the THP-1 cell line to simulate the neovascular microenvironment. The vascularization capacity of HUVECs was examined using the CCK-8 assay kit, tube formation assay, and scratch wound-healing assay. Results: In vivo, the mRNA expression of Beclin-1 and ATG5 was increased, together with the upregulation of M1 macrophage markers (CD86 and INOS) in corneas after early alkali injury. The area of CNV is effectively relieved in the rapamycin-treated mice. In vitro, upregulation of autophagy level by pretreatment with 3-methyladenine (3-MA) could increase the mRNA expression of the M1 markers. Macrophage-conditioned medium with impaired autophagy contains more IL-6 and TNF-α compared to the M1 macrophage-conditioned medium, promoting HUVEC proliferation, migration, and tube formation capacity. Enhancing the autophagy level with rapamycin (RAPA) could reverse this phenomenon. Conclusions: Impaired autophagy promoted macrophage polarization toward M1 type and increased the expression of IL-6 and TNF-α, which led to severe CNV. Using the autophagy activator (RAPA) could effectively alleviate CNV by promoting autophagy.

## 1. Introduction

Corneal neovascularization (CNV) is reported to be a low-grade inflammatory state that is caused by various etiologies such as contact lens wear, chemical burn, limbal stem cell deficiency (LSCD), and ocular surface inflammation [1,2,3]. More than 1.4 million people suffer from CNV annually, which can lead to blindness if they do not receive timely treatment [4,5,6]. CNV is characterized by imbalances between angiogenic and anti-angiogenic factors. Increasing evidence shows that inflammation is the core mechanism of CNV [1,7,8].

As a source of angiogenic factors, macrophages play an important role in the development of inflammation-induced CNV [9]. Early in corneal injury, and before the onset of CNV, polymorphonuclear leukocytes and small numbers of macrophages invade the cornea and participate in the inflammatory response. Macrophages that infiltrate the inflammatory tissue differentiate into functional forms, including classical activation (pro-inflammatory M1) and alternative activation (tissue remodeling and anti-inflammatory M2) macrophages. A significant amount of proangiogenic factors and proteolytic enzymes that promote limbal cell proliferation and migration are produced by inflammation-induced cells, particularly M1 macrophages, when corneal damage occurs [10,11,12]. IL-6 and TNF-α are proinflammatory factors that are overexpressed during keratitis and induce angiogenesis to some extent [13,14,15]. Therefore, understanding the pathogenesis of CNV at the macrophage polarization and inflammatory cytokine secretion levels is essential for the development of effective preventive and therapeutic measures.

Autophagy is a conserved bulk degradation and recycling process by which the cytoplasmic cargo is delivered to the lysosomes for degradation, and it plays an important role in macrophage phagocytosis, antigen presentation, regulating immune response, and inflammatory response [16,17]. Autophagy also serves as a stress response pathway. Increasing evidence suggests that autophagy regulates the polarization direction of macrophages and participates in the process of inflammatory diseases [16,18,19]. Autophagy dysfunction contributes to several diseases, including cancer, inflammation, and autoimmunity. Rapamycin (RAPA) and 3-methyladenine (3-MA), the most widely used autophagic activator and inhibitor, have been reported to affect inflammation [20,21,22]. Regulating autophagy may have the dual role of restoring cell homeostasis and regulating inflammatory signal feedback activation, elucidating the role of autophagy in macrophage polarization in CNV, which not only helps us to understand that macrophages secrete different inflammatory cytokines in each stage but also helps us to provide new ways for the treatment of CNV. Our study aims to explore how autophagy works on inflammation in CNV and whether the mechanism is related to macrophage polarization.

## 2. Methods

### 2.1. Cell Culture

We cultured THP-1 cells (American Type Culture Collection, ATCC, Manassas, VA, USA) in RPMI-1640 complete medium (Gibco, Grand Island, NY, USA) with 10% fetal bovine serum (FBS, Gibco, Grand Island, NY, USA) at 37 °C in a humidified 5% CO_2_ and 95% air atmosphere. For further experiments, THP-1 cells were incubated with 100 ng/mL phorbol 12-myristate 13-acetate (PMA, AdipoGen, San Diego, CA, USA) for 24 h to obtain M0 macrophages. For the autophagy state regulation, M0 macrophages were pretreated with RAPA (RAPA, Selleck Chemicals, Houston, TX, USA) or 3-methyladenine (3-MA, Selleck Chemicals, Houston, TX, USA) for 4 h, and polarized into M1 macrophages with 100 ng/mL lipopolysaccharide (LPS, InvivoGen, San Diego, CA, USA) + 20 ng/mL interferon-γ (IFN-γ, Cyagen, Santa Clara, CA, USA) stimulation for 24 h. Human umbilical vein endothelial cells (HUVECs) from the ATCC were cultured in Dulbecco’s modified eagle medium (DMEM, Gibco, Grand Island, NY, USA) supplemented with 10% FBS, 50 ng/mL gentamicin, and 50 ng/mL amphotericin B at 37 °C in an atmosphere of 95% O_2_ and 5% CO_2_.

### 2.2. M1 Macrophage Polarization from THP-1 Cells and Acquisition of the Conditioned Medium

To obtain M1 macrophages, THP-1 cells were stimulated with 100 mg/mL PMA for 24 h and then cultured with 100 ng/mL LPS + 20 ng/mL INF-γ for 24 h. We collected four macrophage-conditioned mediums (MCM: M0CM, M1CM, RACM, 3MCM). The M0 macrophage-conditioned medium (M0CM) was collected directly for other experiments. To obtain the M1 macrophages with the different autophagy states and other different macrophage-conditioned mediums (M1CM, RACM, 3MCM), M0 cells were pretreated with 100 nM RAPA or 1 mM 3-MA for 4 h, and then treated with LPS and IFN-γ for 24 h. Cells were grown for a further 24 h after medium replacement with fresh DMEM to produce different M1 macrophage-conditioned mediums (defined as an M1 macrophage polarization-conditioned medium: M1CM, RACM, 3MCM), and then 4 sets of MCM were transferred into HUVEC cells for culture and harvested for other experiments. The MCM was centrifuged at 10,000 rpm for 10 min before cultured cells or storage.

### 2.3. Reverse Transcription–Quantitative Polymerase Chain Reaction (RT-qPCR)

Total RNAs were isolated from macrophages and corneal tissue using TRIzol reagent (Invitrogen, Carlsbad, CA, USA) and RNAs were reverse-transcribed into complementary DNAs (cDNAs) by RT-Master-Mix (MedChemExpress, Monmouth Junction, NJ, USA) following the manufacturer’s instructions. As a template for PCR, the cDNA was used with the SYBR-Green-qPCR Master Mix (MedChemExpress, Monmouth Junction, NJ, USA) on an ABI 7500 RT-PCR (Applied Biosystems, Foster City, CA, USA) thermocycler. After relative expressions were normalized to GAPDH, the data were analyzed using the 2^−ΔΔCT^ method. Primers were designed by Sangon Biotech Company (Shanghai, China) and are shown in Table 1.

### 2.4. Western Blot (WB)

Total protein was extracted by using RIPA buffer (Beyotime, Shanghai, China). A total of 20 μg of protein was subjected to SDS-PAGE using 10% polyacrylamide gels (Beyotime, Shanghai, China). Following a 1.5 h block with 5% non-fat milk, membranes were incubated with primary antibodies against Beclin-1 (diluted 1:1500; Abcam, Cambridge, UK), ATG-5 (diluted 1:1500; Abcam, Cambridge, UK) and GAPDH (diluted 1:3000; Cell Signaling Technology, Danvers, MA, USA) overnight at 4 °C. After washing, the membranes were incubated with the HRP AffiniPure Goat anti-rabbit IgG (diluted 1:3000; EarthOx, Millbrae, CA, USA) for 1.5 h. The proteins were visualized using gel densitometry (BioRad Laboratories, Hercules, CA, USA) and analyzed by ImageJ software.

### 2.5. Scratch Wound-Healing Assay for Cell Migration

About 5 × 10^5^ HUVECs were inoculated on 6-well plates and cultured until 100% confluence. After removing free cells by three washes with PBS, HUVECs were treated with conditioned medium and plates were incubated at 37 °C. Images were taken at 0 and 36 h after scratch wounding and analyzed by ImageJ software.

### 2.6. Tube Formation Assay

The basement membrane substrate (Matrigel, BD Biosciences, Franklin Lakes, NJ, USA) was dissolved at 4 °C for 3–5 h before the experiment and then injected into 96-well plates at 100 uL per well. The wells were incubated at 37 °C for 30 min followed by 2 × 10^4^ HUVECs cultured in 200 μL of conditioned medium. Images were acquired for 0, 36 h and the total number of branches, junctions, and segments was calculated using ImageJ software.

### 2.7. Cell Counting Kit-8 (CCK-8) Assay

The proliferation of THP-1 cells and HUEVCs was tested by a CCK8 assay kit (Beyotime, Shanghai, China) according to the manufacturer’s instructions. HUVECs were plated in 96-well culture plates with different macrophage-conditioned mediums (MCMs) and incubated for 3 or 6 h, before the supernatant was updated for another 30 min at 37 °C with RPMI-1640 medium containing 10 μL CCK-8. The optical density (OD) value of each sample was measured at 450 nm using a microplate reader (Thermo Fisher Scientific, Carlsbad, CA, USA).

### 2.8. Enzyme-Linked Immunosorbent Assay (ELISA)

The MCMs were collected from each group and stored at −80 °C. IL-6 and TNF-α concentrations in each group of cultured supernatants were measured by the ELISA kit (R & D Systems, Minneapolis, MN, USA) according to the manufacturer’s protocols. The OD value at 450 nm was detected using the Varioskan LUX multimode microplate reader (Thermo Fisher Scientific, Carlsbad, CA, USA).

### 2.9. Corneal Alkali Burn Model and Rapamycin Treatment

C57 mice (6–8 weeks of age) purchased from the Animal Experiment Center of Chongqing Medical University were randomly assigned to each group. To explore the expression changes in autophagy proteins in the cornea of alkali-burned mice, alkali-burned mice (*n* = 6) and normal control mice (*n* = 6) were included in each replicated experiment. All alkali-burned mice were anesthetized by intraperitoneal injection of 3% sodium pentobarbital (50 mg/kg). Alkali burn injury was inflicted by pressing a 2.0 mm diameter filter paper plate soaked in 1 mM NaOH onto the left and right central cornea of mice for 15 s. The corneal surface and the conjunctival sac were then thoroughly rinsed with 0.9% sodium chloride solution for 2 min. Control mice were continuously observed without requiring any treatment. To verify the therapeutic effect of RAPA, alkali-burned mice (*n* = 6) compared with rapamycin-treated alkali-burned mice (*n* = 6) were included in each replicated experiment. We injected RAPA (10 mg/kg) into the abdominal cavity of the mice starting two days before alkali burn modeling, injected RAPA daily after alkali burn modeling, and added a drop of Rapa (10 mM) to the corneal. CNV was observed with a slit lamp on day 7 and day 14. The study was approved by the Ethics Committee of the First Affiliated Hospital of Chongqing Medical University (no. 2021-003, 16 January 2021) and followed the principles of the Declaration of Helsinki.

### 2.10. Evaluation of Corneal Morphology under Slit Lamp

Corneal neovascular growth was observed, and the cornea was photographed under a slit lamp on days 7 and 14 after mold creation. Neovasculature length perpendicular to the limbus was measured, and area of CNV was calculated following the Robert formula. Cornea neovascular area was calculated as A = C/12 × 3.14 × [R^2^ − (R − L)^2^], where A is the CNV area, C is the number of peripheral hours accumulating in the neovascular cornea, R is the corneal radius of mice, and L is the length of neovasculature extending from the limbus into the cornea.

### 2.11. Statistical Analysis

Each experiment was repeated at least three times. The SPSS 20.0 software package (Chicago, IL, USA) was used for statistical analysis. Statistical significance was assessed using Student’s *t*-test for two-group comparisons or one-way analysis of variance (ANOVA). Mean ± standard deviation (SD) from 3–6 independent experiments was used to display data. A *p*-value under 0.05 was considered to be statistically significant.

## 3. Results

### 3.1. Autophagy Was Activated in Alkali Burn-Induced CNV and M1 Macrophages

**Figure 1:**
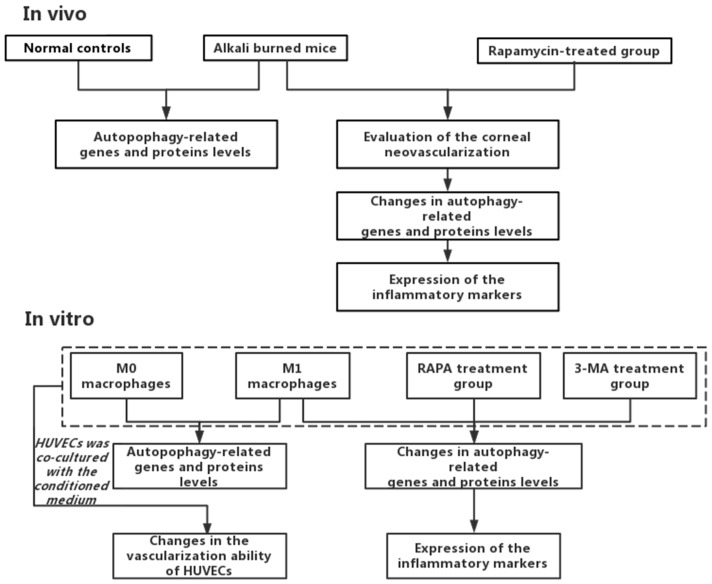
The general route diagram.

**Figure 2:**
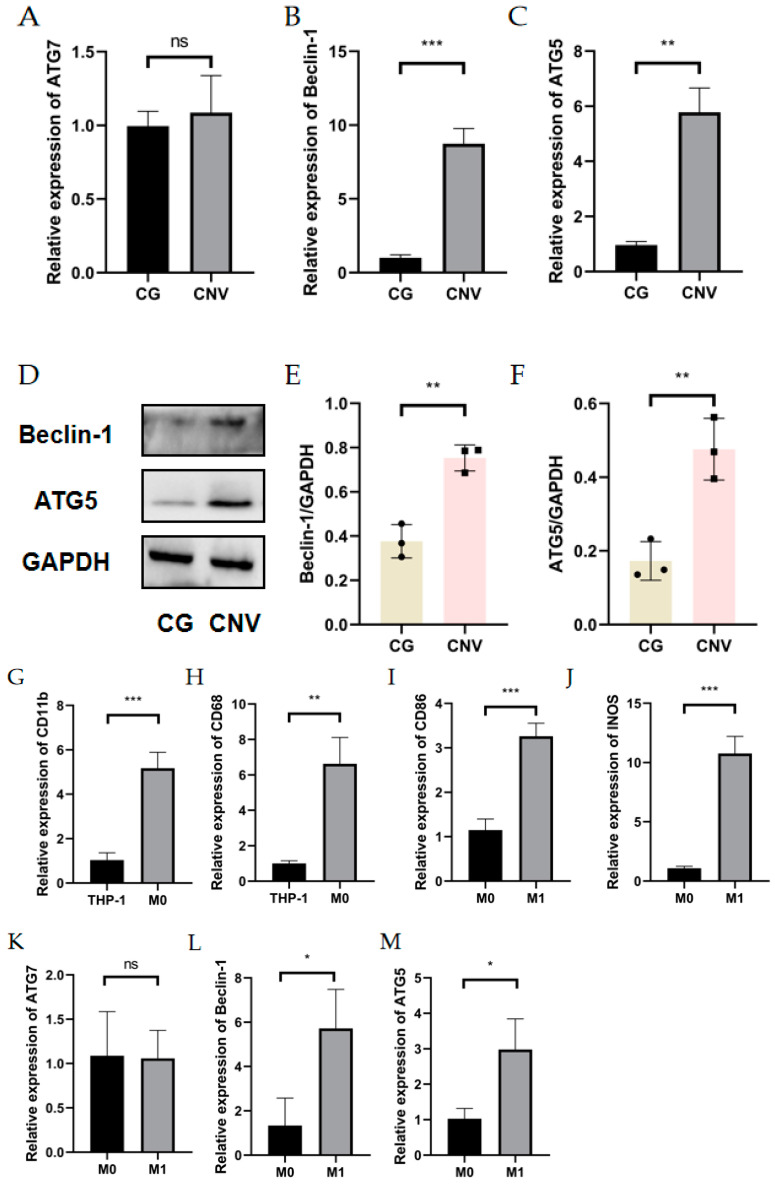

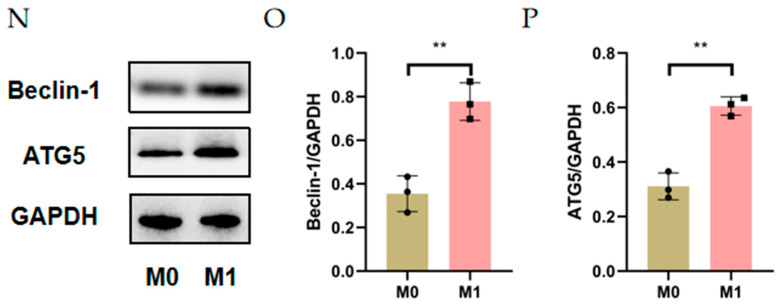
Autophagy level in alkali burn mouse models and macrophage inflammation models. (**A**–**C**) The mRNA expression of autophagy-related genes (ATG7, Beclin-1, ATG5) in the corneal epithelium analyzed by RT-qPCR. *n* = 3:3. (**D**) The changes in autophagy-related protein (Beclin-1 and ATG5) expression in the corneal epithelium after alkali burn treatment analyzed by Western blot. *n* = 3:3. (**E**,**F**) Quantification of the Western blot result for Beclin1 and ATG5 in (**D**). (**G**,**H**) The mRNA expressions of CD11b and CD68 were analyzed by RT-qPCR in THP-1 cell line and M0 macrophages. (**I**,**J**) The mRNA expressions of CD86 and INOS were analyzed by qRT-qPCR in M0 macrophages and M1 macrophages. *n* = 3:3. (**K**–**M**) The mRNA expression of autophagy-related genes (ATG5, ATG7, Beclin-1) in M0 macrophages and M1 macrophages analyzed by RT-qPCR. (**N**) The changes in Beclin1 and ATG5 protein expression in M0 macrophages and M1 macrophages analyzed by Western blot. (**O**,**P**) Quantification of the Western blot result for Beclin1 and ATG5 in (**N**). Unpaired *t*-tests were used for statistical analyses. ns means no significant difference, * *p* < 0.05, ** *p* < 0.01, *** *p* < 0.001.

The general route diagram is shown in Figure 1. To investigate the expression pattern of autophagy during CNV, the expression levels of autophagy-related genes and proteins were examined in alkali burn-induced corneas and LPS- and INF-γ-induced M1 macrophages. In vivo, the mRNA expressions of Beclin-1 and ATG5 were elevated in mouse corneas affected by alkali burns compared with the control group (CG) (Figure 2B,C), whereas the mRNA expression of ATG7 showed no significant difference (Figure 2A). Western blot showed elevated protein levels of Beclin-1 and ATG5 compared to the controls (Figure 2D–F). In vitro, the RT-qPCR results showed significantly higher expression of the M0 markers (CD11b, CD68) (Figure 2G,H) in M0 macrophages. The results of RT-qPCR showed that the expressions of M1 macrophage markers (CD86, INOS) were significantly elevated (Figure 2I,J), indicating successful induction of M1 macrophages. The mRNA and protein expression of Beclin-1 and ATG5 were increased in M1 macrophages compared to M0 macrophages (Figure 2L–P). These data imply that both CNV and macrophage polarization toward the M1 phenotype can promote autophagy activation.

### 3.2. Enhanced Autophagy Suppresses M1 Macrophage Markers and Impaired Autophagy Enhances M1 Macrophage Markers

**Figure 3:**
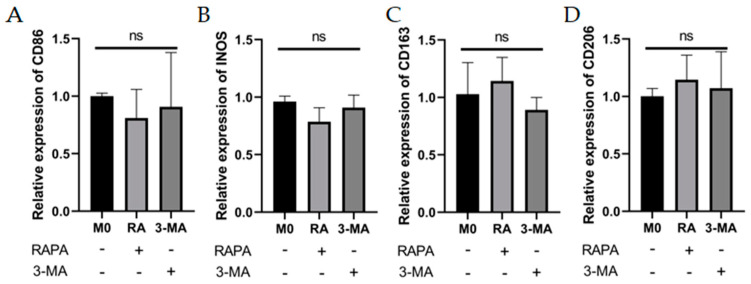

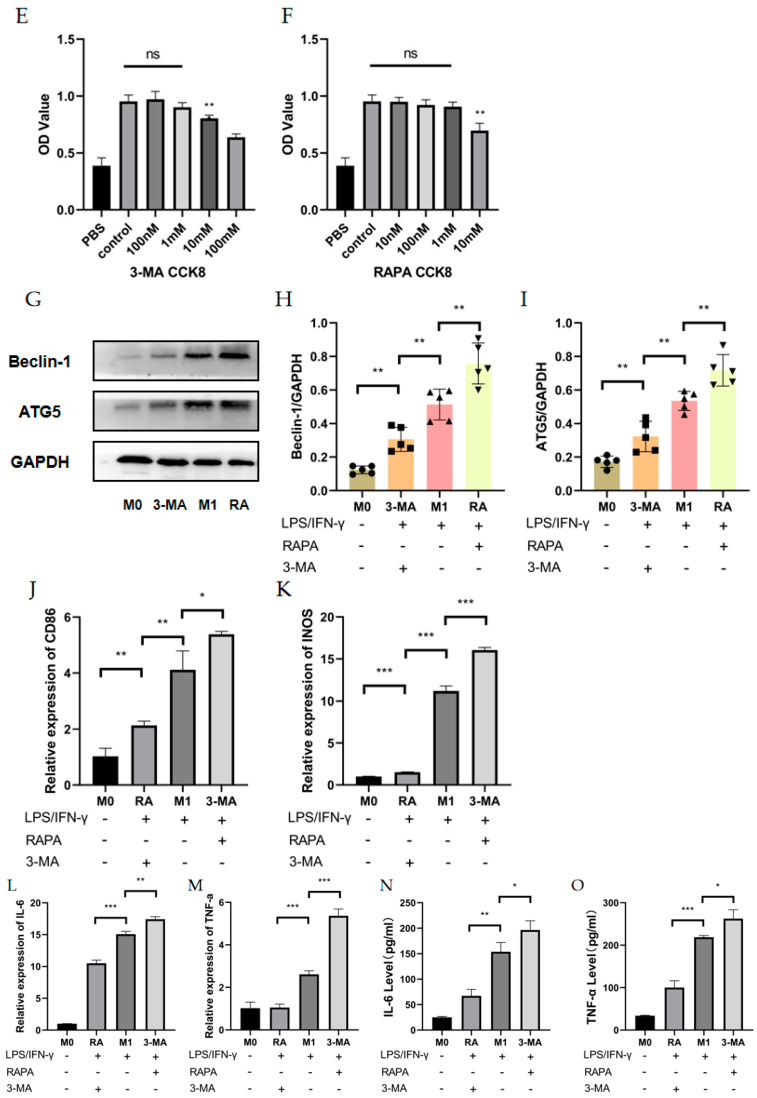
Effects of autophagy levels on macrophage polarization and inflammation-related cytokines. (**A**–**D**) The mRNA expression of M1 macrophage markers (CD86 and INOS) and M2 macrophage markers (CD163 and CD206) were analyzed by RT-qPCR in M0 macrophages after RAPA (100 nM) or 3-MA (1 mM) treatment. *n* = 6:6. (**E**,**F**) The cell viability of THP-1 cells after pretreatment with RAPA (10 nM, 100 nM, 1 mM, 10 mM) and 3-MA (100 nM, 1 mM, 10 mM, 100 mM) for 4 h analyzed by CCK8 assay. *n* = 6:6. (**G**) The changes in Beclin-1 and ATG5 protein expression in M0 group, RAPA group, M1 group, and 3-MA group analyzed by Western blot. *n* = 5:5:5:5. (**H**,**I**) Quantification of the Western blot result for Beclin-1 and ATG5 in (**G**). (**J**,**K**) The mRNA expression of CD86 and INOS in M0 group, RAPA group, M1 group, and 3-MA group analyzed by RT-qPCR. *n* = 5:5:5:5. (**L**,**M**) The mRNA expression of IL-6 and TNF-α in M0 group, RAPA group, M1 group, and 3-MA group analyzed by RT-qPCR. (**N**,**O**) IL-6 and TNF-α protein expression analyzed by ELISA in M0 group, RAPA group, M1 group, and 3-MA group. *n* = 6:6:6:6. ANOVA was used to analyze the variance in multiple groups. Unpaired *t*-test was used to analyze the variance between groups. ns means no significant difference, * *p* < 0.05, ** *p* < 0.01, *** *p* < 0.001.

The RT-qPCR results showed no statistically significant effects of RAPA or 3-MA treatment on the M1 (CD86, INOS) and M2 macrophage markers (CD163, CD206) (Figure 3A–D) in M0 macrophages. For M1 macrophages, M1 macrophage-associated markers would be attenuated with increased expression of the autophagy-related proteins (Beclin-1, ATG5), whereas diminished autophagy would give the opposite result. The CCK-8 assay showed that the pretreatment of THP-1 cells with 10 nM–1 mM RAPA and 100 nM–1 mM 3-MA had no significant effect on the cell activity for 4 h (Figure 3E,F). The pretreatments of 100 nM RAPA and 1 mM 3-MA were chosen to establish the regulation of the autophagy model. WB results showed that we successfully regulated the degree of autophagy (Figure 3G–I). Interestingly, 3-MA increased the mRNA expression of INOS and CD86, while RAPA suppressed the increase in M1 mRNA levels in LPS- and IFN-γ-induced M1 macrophages (Figure 3J,K). Detection of IL-6 and TNF-α expression levels at mRNA by RT-qPCR and detection of cytokine levels in cell secretion by ELISA found that the autophagy activator (RAPA) reduced IL-6 and TNF-α expression at mRNA and protein levels, while the autophagy inhibitor (3-MA) enhanced TNF-α and IL-6 expression in M1 macrophages (Figure 3L–O).

These data imply that passive autophagy changes are not sufficient to drive macrophage polarization in some directions, but that in inflammatory states, the inflammatory factors IL-6 and TNF-α have opposite trends with autophagy levels at both gene and protein levels. Autophagy inhibition can promote macrophage polarization toward the M1 phenotype and autophagy activation acts in an opposite manner under inflamed status, but improving the degree of autophagy did not restore the inflammatory factor to the inflammatory resting state.

### 3.3. The Culture Supernatant of Impaired M1 Macrophages Enhanced the Proliferation, Migration, and Tube Formation Capacity of HUVECs

**Figure 4:**
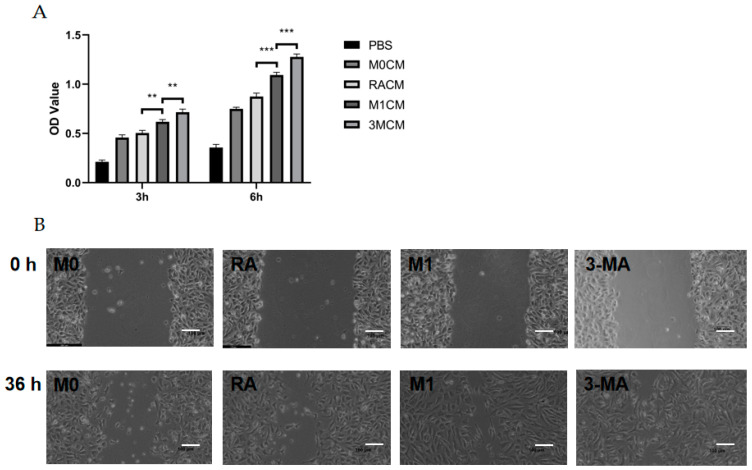

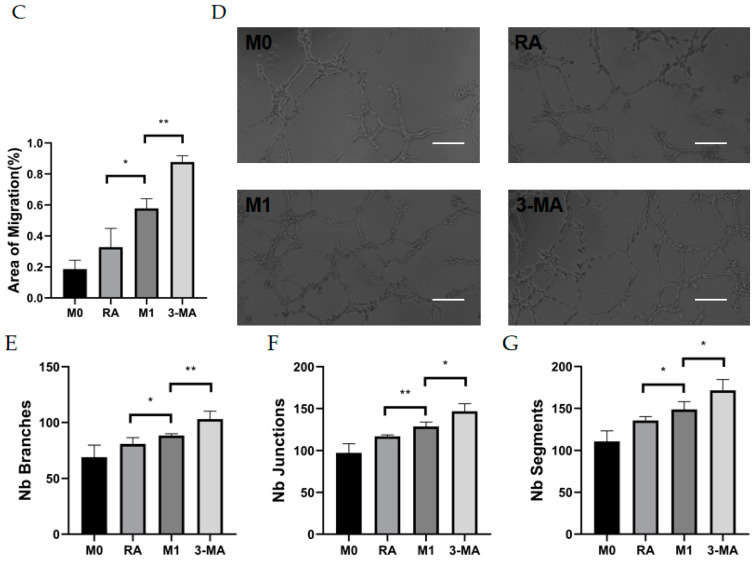
Vascularization ability of HUVECs after co-culture with culture supernatants of macrophages with different autophagy states. (**A**) The cell viability of HUVECs after co-culture with different culture supernatants (MOCM, RACM, M1CM, 3MCM) for 3 h to 6 h analyzed by CCK8 assay. *n* = 6:6:6:6. (**B**) Representative images of HUVECs scratch wound assay at 0 h and 36 h. Scale bar = 100 μm. (**C**) Statistical analysis of HUVECs migration distance. *n* = 3:3:3:3. (**D**) Representative images of the tube formation of HUVECs after co-culture with different culture supernatants (MOCM, RACM, M1CM, 3MCM) for 6 h. Scale bar = 100 μm. (**E**–**G**) Statistical analysis of HUVECs tube formation, including the number of branches, junctions, and segments. *n* = 6:6:6:6. ANOVA was used to analyze the variance in multiple groups. Unpaired *t*-test was used to analyze the variance between groups. * *p* < 0.05, ** *p* < 0.01, *** *p* < 0.001.

To investigate the relationship between CNV and macrophage autophagy, we collected M0 macrophage culture supernatant (M0CM group), M1 macrophage culture supernatant (M1CM group), autophagy-enhanced M1 macrophage culture supernatant (RACM group), and autophagy-impaired M1 macrophage culture supernatant (3MCM group), as described above. After co-culture of HUVECs with the four conditioned media, the proliferative capacity of the HUVECs was enhanced by M1 macrophage culture supernatant (M1CM group) at 3 h and 6 h compared with M0 macrophage culture supernatant (M0CM group). Compared with the M1CM group, the autophagy-impaired culture supernatant group (3MCM group) significantly promoted the HUVECs’ proliferation (Figure 4A). The scratch wound-healing assay showed the scratch areas of the four groups at 0 h and 36 h, and the results indicated the highest migration distance and the highest mobility in the 3MCM group (Figure 4B,C). Similar results were obtained from the tube formation assay, and the number of branches, junctions, and segments of the HUVECs increased in the M1CM group compared to the M0CM group. The data from the 3MCM group demonstrated a further increase after impaired autophagy (Figure 4D–G). Interestingly, before the polarization of M0 macrophage to M1, we enhanced autophagy using RAPA pretreatment as compared to the M1CM group and 3MCM group, finding that HUVECs’ proliferation, migration, and tube formation capacity would be inhibited (Figure 4D–G). The above experiments show that impaired autophagy is indeed an important factor in inflammation-induced CNV, and impaired autophagy exacerbated inflammation and thus enhanced the proliferation, migration, and tube formation capacity of HUVECs.

### 3.4. Autophagy Enhancement Improved Corneal Neovascularization in Alkali Burned Mice

**Figure 5:**
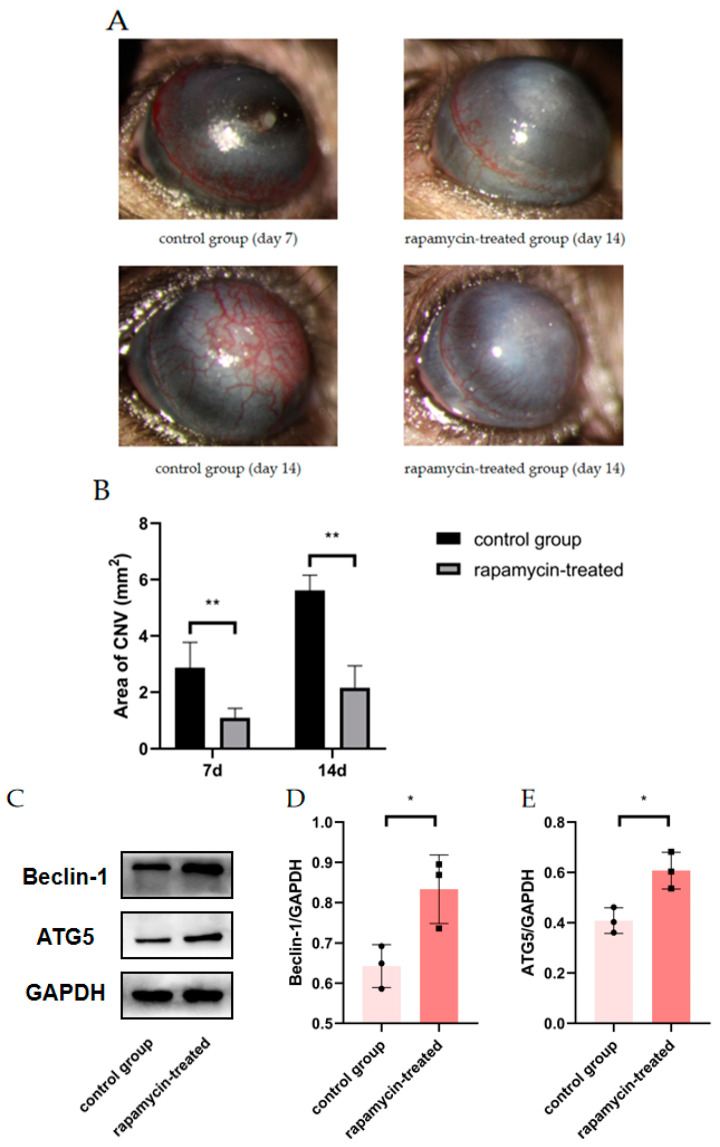

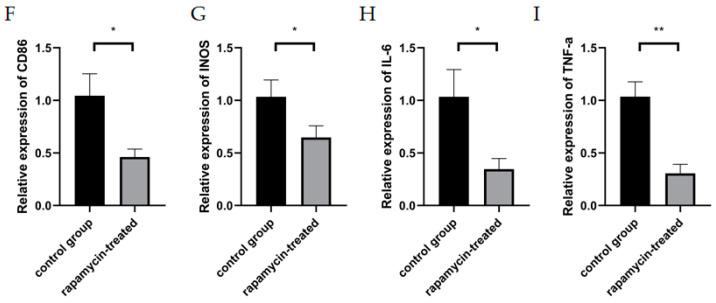
The effect of RAPA treatment on CNV. (**A**) Representative images of alkali-burned mice treated with RAPA on day 7 and day 14. (**B**) Quantification of CNV area in the rapamycin-treated group and the control group on day 7 and day 14. *n* = 6:6. (**C**) The changes in Beclin1 and ATG5 protein expression in the corneas of untreated alkali burned mice and those of rapamycin-treated alkali burned mice analyzed by Western blot. (**D**,**E**) Quantification of the Western blot result for Beclin1 and ATG5 in (**C**). *n* = 3:3. (**F**–**I**) mRNA expression of M1 macrophage markers (INOS, CD86, IL-6, TNF-α) analyzed by RT-qPCR in mouse corneas. *n* = 3:3. Unpaired *t*-test was used to analyze the variance between groups. * *p* < 0.05, ** *p* < 0.01.

To confirm whether mouse CNV could be alleviated by improving autophagy, alkali burn mice were treated with RAPA. We found a significant reduction in CNV area in alkaline burn mice after RAPA injection, as compared to untreated alkaline burn mice (Figure 5A,B). WB demonstrated the efficacy of RAPA in enhancing macrophage autophagy (Figure 5C–E). The RT-qPCR revealed that the inflammatory markers (INOS, CD86, IL-6, and TNF-α) were significantly reduced in the corneas of RAPA-treated mice compared with alkali burn mice (Figure 5F–I). Thus, we found that RAPA reduces inflammatory cytokine expression and relieves CNV in alkali-burned mice.

## 4. Discussion

The cornea is considered to maintain avascular transparent tissue but can induce CNV in various pathological conditions. Inflammation is the major pathological process in CNV formation and involves complex cellular interactions and chemotactic signaling [1]. Macrophage polarization has been the focus of inflammation. M1 macrophages as proinflammatory cells have an important role in inflammation-related diseases. Physiological corneal vascular supply is maintained by the net balance between proangiogenic and anti-angiogenic factors, corneal damage induced by various growth factors, cytokines, and chemokines, all in a coordinated way to promote tissue repair [23,24]. However, excessive secretion of some inflammatory factors breaking this balance will also cause more severe pathological damage. Immature blood vessels mainly rely on angiogenic factors and inflammatory mediators for proliferation and growth, in which macrophage polarization plays a crucial role [25,26]. Autophagy plays an important role in macrophage phagocytosis, antigen presentation, regulating immune response, and inflammatory response. It can also regulate the polarization direction of macrophages and participate in the process of inflammatory diseases such as sepsis, acute lung injury, inflammatory liver injury, atherosclerosis, and rheumatoid arthritis. Autophagy is present in all the different stages of inflammation, ranging from macrophage differentiation and polarization to the secretion of inflammatory mediators and cytokines [27,28,29]. Regulating autophagy may have the dual role of restoring cell homeostasis and regulating inflammatory signal feedback activation, elucidating the role of autophagy in macrophage polarization in CNV, which not only helps us to understand that macrophages secrete different inflammatory cytokines at different stages but also helps us to provide new ways for the treatment of CNV in different mature stages. Thus, the present study sought to understand the mechanisms of CNV formation through the effects of autophagy on macrophage polarization. Our study found that autophagy-impaired macrophages would disrupt this balance for more severe CNV, but rather for some remission if we could use RAPA early in inflammation.

This study used alkali burn-induced mice and an LPS- and IFN-γ-stimulated macrophage model. Costimulation of LPS and IFN-γ is a conventional method to induce M0 polarization to M1 [30]. We examined the mRNA expression levels of the M1 macrophage markers (CD86 and INOS). We found that CD86 and INOS expressions were elevated after 24 h of coinduction of LPS and IFN-γ, indicating that M1 macrophages were successfully induced.

Autophagy is a process that phagocytoses own organelles or cytoplasmic proteins. The substance to be phagocytosed is coated with lysosomes to form autophagysosomes to degrade its coated contents, thereby achieving the metabolic needs of the cell itself and the renewal of certain organelles [31]. Autophagy has been identified as an important factor in the inflammatory response and immune cells. We examined the expression levels of Beclin-1 and ATG5 in THP-1 cells treated with LPS and IFN-γ for 24 h to assess the role of autophagy in M1 macrophage activation. The expression levels of both autophagy-related genes and proteins were significantly increased in M1 macrophages as compared to unactivated M0 macrophages, perhaps as a result of accelerated inflammatory vesicle motility and organelle renewal [32,33]. Although autophagy proteins alone are not enough to initiate or silence inflammation, regulating autophagy and reactivating M0 macrophage polarization to M1 macrophages, we found that autophagy can inhibit M1 macrophage polarization, and instead, impaired autophagy will lead to more severe inflammation. Here, we clearly show that LPS and IFN-γ promote autophagy in macrophages as studies progressed, while the expression levels of ATG5 and Beclin-1 are contrary to the expression trend of M1 markers INOS and CD86, indicating that autophagy may be involved in the regulation of M1 macrophage activation and may inhibit inflammation overdevelopment. Although we did not find in vitro that autophagy can promote the polarization of THP-1 cell lines toward M1 or M2, CNV causes impaired autophagy and develops a strong inflammatory response, suggesting that the autophagic dysfunction in macrophages may lead to a disordered inflammatory response in CNV.

Angiogenesis is a complex process, particularly associated with vascular endothelial cells. Endothelial cell proliferation and migration are prerequisites for capillary formation. Chemokines and proinflammatory cytokines are overexpressed in corneal inflammation and contribute to promoting angiogenesis [34,35]. Alkali burns increased the expression of IL-6 and TNF-α, genes that play a key role in CNV. The polarization of macrophages to M1 type will release inflammatory factors such as IL-6, TNF-α, and IL-1. Among these pro-angiogenic molecules, IL-6 is a pro-inflammatory cytokine produced in corneal chemical burn tissue, which is known to induce the production of angiogenic factors to promote CNV [11,13,36]. TNF-α, which is mainly synthesized by macrophages, also plays a key role in inflammatory diseases, increasing the expression of adhesion molecules and inflammatory mediators in human corneal epithelial cells, and recruiting leukocytes by producing chemokines [37]. Indeed, high levels of IL-6 and TNF-α accumulate substantially in the early alkali burn cornea, subsequently accelerating the progression of the inflammatory state, and eventually inducing the invasion of vascular endothelial cells into the transparent cornea. Our study found that IL-6 and TNF-α expression were not only increased in activated macrophages but also further increased in M1 macrophages with impaired autophagy, causing more severe CNV.

IL-6 and TNF-α induce the migration of macrophages and vascular endothelial cells by both enhanced chemokine production and the expression of endothelial cell adhesion molecules [15,37]. We examined the expression patterns of neovascularization-associated growth factors and cytokines in M1 macrophages of varying degrees of autophagy, using RT-qPCR and ELISA, respectively. While autophagy is promoted by RAPA, pro-inflammatory factors such as IL-6 and TNF-α are also inhibited. This effect was inversely validated after the use of the 3-MA. In a model of LPS- and IFN-γ-induced macrophage inflammation, increased autophagy levels inhibited the production of cytokines such as pro-inflammatory TNF-α, IL-6, IL-1, and IL-12 by promoting autophagy. Our study demonstrates that M1 macrophage polarization is activated by stress caused by alkaline burns. Autophagy can inhibit the inflammatory cytokines IL-6 and TNF-α, and impaired autophagy will promote the production of more severe inflammation. By cultivating HUVECs with autophagy, through CCK-8 and scratch experiments, we can see that HUVECs will obtain stronger proliferation and migration capacity, and under the validation of the tube formation assay, we demonstrated that HUVECs achieved more vessel-forming capacity, which can alleviate this phenomenon to some extent. The inhibition of CNV by autophagy may be due to the downregulation of M1 macrophage polarization, and autophagy regulates the expression of proinflammatory factors IL-6 and TNF-α by playing a key role [38,39]. Our present study determined that autophagy can partly regulate the immune response by affecting M1 polarization. Recent studies have demonstrated an important role of autophagy in the sequestration and clearance of pathogens. Autophagy plays an essential function in innate immunity by recruiting monocytes, regulating the differentiation of macrophages and the fate of hematopoietic stem cells [40,41]. However, in our study, the anti-inflammatory function of autophagy was demonstrated in the alkali burn mice model and the in vitro model of inflammation.

There is increasing evidence that many diseases are caused by an excessive immune response, in which autophagy plays a regulatory function. Autophagy plays a stable and balanced role by regulating immune cell responses to various stimuli, especially in anti-inflammatory processes [42,43]. The degree of tissue inflammation induced by the innate immune response is largely dependent on the balance between the macrophages being polarized to the pro-inflammatory M1 and the anti-inflammatory M2 macrophages [44]. The uninhibited M1 macrophage response is associated with prolonged tissue damage, but the present results suggest that impaired M1 macrophage autophagy may be involved in the process of increased inflammation and tissue damage.

Accordingly, we hypothesized that impaired autophagy may have negative effects on CNV by enhancing the promotion of macrophages. The role of autophagy in inflammation and immune responses has been gradually elucidated in various fields, enabling autophagy-related genes and proteins to become therapeutic targets for CNV and other inflammation-related diseases.

## Figures and Tables

**Table 1:** Table of primers.

Primer Name	5′->3′ Forward	5′->3′ Reverse
mice GAPDH	GAGAGTGTTTCCTCGTCCC	ATTTGCCGTGAGTGGAGTC
mice Beclin-1	GGGGGTTTGCGGTTTTTCTG	CTGCCTCCAGTGTCTTCAATCT
mice ATG5	TATCAGACCACGACGGAGC	CAGAGGGGTTTCCAGCATTG
mice ATG7	CCAACTCCACACTGCTTTCCT	CTACTGTTCTTACCAGCCTCACT
mice CD86	TCAGTATCTCCAACAGCCTCTC	TCCAGAACACACACAACGGT
mice INOS	GCCACCTCTACATTTGCGGA	CTGCTCCTCGCTCAAGTTCA
mice IL-6	GCCTTCTTGGGACTGATGCT	GGTCTGTTGGGAGTGGTATCC
mice TNF-α	GAGAAGGGGGACCAACTCAG	CTCCAAAGTAGACCTGCCCG
human GAPDH	ATCCCATCACCATCTTCCAGG	AAATGAGCCCCAGCCTTCTC
human Beclin-1	GAGGTTGAGAAAGGCGAGACA	GAGGACACCCAAGCAAGACC
human ATG5	CGAGATGTGTGGTTTGGACGA	TGGTTCTGCTTCCCTTTCAGTT
human ATG7	AACCTCTCTTGGGCTTGTGC	GGCTGACGGGAAGGACATT
human CD11B	TATGACTGGGCTGGTGGAGT	TCTGCCTGAACATCGCTACC
human CD68	ACTGAACCCCAACAAAACCA	GGAATGAGAGAAGCAGGTGG
human INOS	GATGGGAGAAGGGGATGAGC	GAATGTGCTGTTTGCCTCGG
human CD86	CCCCAGACCACATTCCTTGG	TGTTCACTCTCTTCCCTCTCCA
human CD163	GCTCAGGAAACCAGTCCCAA	TACCAGGCGAAGTTGACCAC
human CD206	TACTGAACCCCCACAACTGC	ACCAGAGAGGAACCCATTCG
human IL-6	CTTCGGTCCAGTTGCCTTCT	GGTGAGTGGCTGTCTGTGTG
human TNF-α	ACCTCCTCTCTGCCATCAAGA	TCCCAAAGTAGACCTGCCCA

## Data Availability

Not applicable.

## Abbreviations

CNV	Corneal neovascularization	HUVECs	Human umbilical vein endothelial cells
RT-qPCR	Real-time quantitative PCR	CCK-8	Vecsxaz Cell Counting Kit-8
ELISA	Enzyme-Linked Immunosorbent Assay	ATG5	Autophagy-related 5
ATG7	Autophagy-related 7	INOS	Inducible nitric oxide synthase
GAPDH	Glyceraldehyde-3-phosphate dehydrogenase	IL-6	Interleukin-6
TNF-α	Tumor necrosis factor-alpha	RAPA	Rapamycin
3-MA	3-methyladenine	LPS	Lipopolysaccharide
INF-γ	Interferon-gamma	ATCC	American Type Culture Collection
FBS	Fetal bovine serum	PMA	Phorbol 12-myristate 13-acetate
LSCD	Limbal stem cell deficiency	DMEM	Dulbecco’s modified eagle medium
M0CM	M0 macrophage-conditioned medium	MCM	Macrophage-conditioned medium
M1CM	M1 macrophage-conditioned medium with no pretreatment	RACM	M1 macrophage-conditioned Medium with rapamycin pretreatment
3MCM	M1 macrophage-conditioned medium with 3-MA pretreatment	CG	Control group
